# Impact of LAB from Serpa PDO Cheese in Cheese Models: Towards the Development of an Autochthonous Starter Culture

**DOI:** 10.3390/foods12040701

**Published:** 2023-02-06

**Authors:** Helena Araújo-Rodrigues, António P. L. Martins, Freni K. Tavaria, João Dias, Maria Teresa Santos, Nuno Alvarenga, Manuela E. Pintado

**Affiliations:** 1 CBQF—Centro de Biotecnologia e Química Fina—Laboratório Associado, Escola Superior de Biotecnologia, Universidade Católica Portuguesa, Rua Diogo Botelho 1327, 4169-005 Porto, Portugal; 2 Instituto Nacional de Investigação Agrária e Veterinária, Avenida da República, Unidade de Tecnologia e Inovação, Quinta do Marquês, 2780-157 Oeiras, Portugal; 3 Geobiosciences, Geobiotechnologies and Geoengineering (GeoBioTec), Faculdade de Ciências e Tecnologias, Universidade Nova de Lisboa, 2829-516 Caparica, Portugal; 4 Instituto Politécnico de Beja, Escola Superior Agrária, Rua Pedro Soares, 7800-295 Beja, Portugal

**Keywords:** Serpa PDO cheese, Lactic acid bacteria, autochthonous starter culture, cheese models, proteolysis, lipolysis

## Abstract

Serpa is a protected designation of origin (PDO) cheese produced with raw ewes’ milk and coagulated with *Cynara cardunculus*. Legislation does not allow for milk pasteurization and starter culture inoculation. Although natural Serpa’s rich microbiota allows for the development of a unique organoleptic profile, it also suggests high heterogeneity. This raises issues in the final sensory and safety properties, leading to several losses in the sector. A possible solution to overcoming these issues is the development of an autochthonous starter culture. In the present work, some Serpa cheese Lactic acid bacteria (LAB)-isolated microorganisms, previously selected based on their safety, technological and protective performance, were tested in laboratory-scale cheeses. Their acidification, proteolysis (protein and peptide profile, nitrogen fractions, free amino acids (FAA)), and volatiles generation (volatile fatty acids (VFA) and esters) potential was investigated. Significant differences were found in all parameters analyzed, showing a considerable strain effect. Successive statistical analyses were performed to compare cheese models and Serpa PDO cheese. The strains *L. plantarum* PL1 and PL2 and the PL1 and *L. paracasei* PC mix were selected as the most promising, resulting in a closer lipolytic and proteolytic profile of Serpa PDO cheese. In future work, these inocula will be produced at a pilot scale and tested at the cheese level to validate their application.

## 1. Introduction

Most Portuguese Protected Designation of Origin (PDO) cheeses using ewes’ raw milk share a standard production method. It includes the following steps: coagulation, primarily using an aqueous extract of cardoon flower; dehydration, via the removing of the whey after cutting and working the curd; molding, mostly handmade using cylindrical shapes; salting, via addition to the milk during filtering; and working the curd or rubbing on the curd surface after pressing [1]. These steps influence the features of the final product. However, most biochemical changes occur during ripening due to environmental conditions [2] and intrinsic microbiota [1,3]. Although Portuguese PDO cheeses including Serpa, Serra da Estrela, Castelo Branco, and Azeitão share a similar production process, these are produced in different geographic areas. Beyond geographical location, animal breed [4], feedstock, season [1], milking [5], or environmental conditions inside the ripening rooms [6] are some factors impacting cheese microbiota. Thus, these parameters strongly influence biochemical changes during ripening and the final product profile [7].

Serpa PDO cheese is produced in the southern Alentejo region with raw ewes’ milk and using an aqueous extract of *Cynara cardunculus* L. as a coagulant. Its ripening period ranges from 30 to 40 days [8], with a minimum of 30 days required by PDO specifications [5,9,10,11]. The economic and cultural impact of Serpa cheese results from their long-lasting cultural heritage concerning the cheesemaking process and sensory profile [4,9]. The preference for this PDO cheese is based mainly on organoleptic attributes, namely a characteristic strong flavor coupled with a semi-soft and creamy texture [4,5,12,13,14].

According to legislation [15], in Serpa PDO cheese, pasteurization and starter culture inoculation are prohibited. This fact results in a complex microbial community mainly arising from raw milk, cardoon flower extract, and the dairy environment [4,12]. However, using raw milk causes a significant heterogeneity in the native microbiota, leading to uncertainty regarding food safety and ripening evolution. Therefore, considerable variability in the final product is registered, resulting in flavor, texture, and safety shortcomings [12,16,17]. The safety risks arising from raw milk use during the cheesemaking process are related to the eventual presence of foodborne pathogens in the complex native microbiota [16,18,19,20] In previous literature data, the presence of some pathogenic species, such as coagulase positive *Staphylococcus*, was reported in Serpa cheeses, although at an acceptable concentration for this type of product [5].

A possible solution to minimize or overcome these issues is the development of an autochthonous starter culture isolated from the original product [17,18,20,21]. A tailor-made starter culture contributes to higher quality and safety standards. It allows these new products to maintain some of Serpa PDO cheese’s authenticity and to reach more restricted markets in terms of safety regulations [4]. In addition, it is an opportunity to maximize the use of raw milk in the small ruminant sector beyond raw milk with an exceptional microbiological profile [17,22]. However, the difficulty in developing an autochthonous starter culture depends on the proper selection of microorganisms, allowing the development of the typical organoleptic profile [17,23].

In recent decades, cheese models have been used to prospectively study the effect of several technological factors on cheese properties. Tavaria et al. [24] used sheep milk curdled with *Cynara cardunculus* L. flower extract as a Serra da Estrela and similar cheese model. In this study, the amino acid catabolism and the generation of volatiles by lactic acid bacteria (LAB), namely four isolates individually: *Lactobacillus*, *Leuconostoc*, *Lactococcus,* and *Enterococcus*, were investigated. Another example of the same cheese corresponds to Tavaria et al. [20]. The authors studied the technological performance of some strains isolated from Serra da Estrela cheese in cheese model systems. A miniature cheese model prepared under controlled microbiological conditions was also developed by Milesi et al. [25]. The study explored the effect of cultures and enzymes on cheese biochemistry in similar conditions of the food matrix. Moreover, Pereira et al. [26] used a procedure similar to traditional Portuguese cheesemaking. The study investigated the effects of rennet and starter culture on proteolysis. Recently, other authors tested autochthonous *Lactococcus lactis*, *Lacticaseibacillus paracasei,* and *Leuconostoc pseudomesenteroides* from Pico cheese as starter cultures. These were tested in a small-scale pasteurized cheese to evaluate the possible improvement of the manufacturing of Pico cheese [27].

Cheese microbiota (starter and nonstarter) and their secreted enzymes impact the volatile and non-volatile macromolecules in the cheese matrix and their sensorial properties [20,24]. Over ripening, lipolysis, proteolysis, glycolysis, and biochemical modifications are key mechanisms producing organoleptic metabolites and their precursors [24,28]. For example, free amino acids (FAA) are precursors of flavor-related compounds (e.g., isoleucine—Ile, leucine—Leu, valine—Val, phenylalanine—Phe) [29,30]. In contrast, short-chain fatty acids (SCFA) play important roles in aroma features (e.g., butyric—C_4_, iso-butyric—iC_4_, propionic—C_3_) [29]. Other molecules also impact the final organoleptic profile and authenticity, including, for example, medium-chain fatty acids, esters, and peptides [28,29]. A screening of sensory, physical, and chemical parameters of Serpa cheeses produced in different seasons and by distinct producers was carried out by Araújo-Rodrigues et al. [31]. This study proposed a group of FAA, SCFA, volatile fatty acids (VFA), and esters as the most common compounds in Serpa cheese. Among these compounds are glutamic acid (Glu), alanine (Ala), Leu, Val, and Phe, lactic and acetic acid, iC_4_, iso-pentanoic acid (iC_5_), hexanoic acid (C_6_), dodecanoic acid (C_12_), ethyl butanoate, decanoate, and dodecanoate.

In a previous study, *Lactobacillus*, *Lactococcus*, *Leuconostoc,* and *Enterococcus* were isolated and identified in Serpa PDO cheeses at the end of the ripening stage [13]. Between this set of strains, some LAB strains were selected as acid-tolerant by Ruiz-Moyano et al. [14]. In the following study, Araújo-Rodrigues et al. [22] studied some of these LAB, which were generally recognized as safe (GRAS), to evaluate their protective and technological potential for developing an autochthonous starter culture. In the reported study, *Lactiplantibacillus plantarum* PL1 and PL4 strains were suggested as the best candidates for a possible autochthonous starter culture. These results were based on the strains’ proteolytic and lipolytic activities, higher acidification potential, lower D-lactic acid production, and better adaptation to salt and temperature during the Serpa cheesemaking process. Additionally, *L. plantarum* PL1 demonstrated a high antimicrobial effect against pathogenic bacteria. A lower acidification capacity was observed with the *Lacticaseibacillus paracasei* strain. However, combined with other more acidifying strains, this could also be a candidate due to its technological performance [22]. Regardless, these autochthonous strains should be tested in a cheesemaking process to complement this study. 

Therefore, this study aimed to test the best LAB candidates, previously selected by Araújo-Rodrigues et al. [22], for a Serpa cheese autochthonous starter culture on a laboratory scale using cheese models. The effect of these strains on the most critical parameters impacting the sensory properties of cheese will be investigated. These parameters include the main chemical and biochemical changes during ripening (e.g., lipolysis and proteolysis). The relationship between inoculated LAB strains and organoleptic chemical markers such as VFA, peptides, and FAA profiles of cheese models will be evaluated.

## 2. Materials and Methods

### 2.1. Chemicals

Raw ewes’ milk from the Lacaune sheep breed, produced in the demarked area of Serpa cheese, was kindly provided by Nuno Cavaco (Produtos Alimentares, Unipessoal, Lda, Beja, Portugal). In addition, *C. cardunculus* produced in the southern Alentejo region, was provided by the Escola Superior Agrária, Instituto Politécnico de Beja (Beja, Portugal).

The microbiological media used were supplied by Biokar Diagnostics (Allonne, France), namely plate count (PC) agar, De Man, Rogosa and Sharpe (MRS) agar, M17 agar, and rose bengal chloramphenicol agar (RBCA); Merck (Darmstadt, Germany) supplied the Baird-Parker agar (BPA); VWR chemicals (Darmstadt, Germany) supplied the violet red bile glucose agar (VRBGA); and Thermo Fischer Scientific (Waltham, USA) supplied the peptone.

For volatile analysis, 3-octanol was supplied by Merck, while fatty acids (iC_4_, C_4_, iC_5_, pentanoic acid—C_5_, C_6_, heptanoic acid—C_7_, octanoic acid—C_8_, nonanoic acid—C_9_, pentanoic acid—C_10_ and C_12_) and esters (ethyl acetate, ethyl butyrate, ethyl isovalerate, ethyl valerate, ethyl hexanoato, ethyl heptanoato, ethyl octanoato, ethyl nonanoato, ethyl decanoato and ethly dodecanoato) were provided by Sigma-Aldrich (St. Louis, MO, USA). Trichloroacetic acid (TCA) and phosphotungstic acid (PTA) were supplied by Sigma-Aldrich. Regarding FAAs analysis, perchloric acid, AA standards (homoserine, norvaline, aspartic acid—Asp, Glu, cysteine—Cys, asparagine—Asn, serine—Ser, histidine—His, glutamine—Gln, threonine—The, arginine—Arg, Ala, tyrosine—Tyr, Val, methionine—Met, tryptophane—Trp, Phe, Ile and Leu), o-Phthalaldehyde, Benzene-1,2-dicarboxaldehyde (OPA) and o-Phthalic dicarboxaldehyde were obtained from Sigma-Aldrich. The proteins and peptides standards were purchased from GE Healthcare (Clinton, IL, USA), specifically, Thyroglobulin (669 kDa), Ferritin (440 kDa), Aldolase (158 kDa), Conalbumin (75 kDa), Ovalbumin (43 kDa), Carbonic anhydrase (29 kDa), Ribonuclease A (13.7 kDa), and Aprotinin (6.5 kDa), while an antihypertensive peptide (KGYGGVSLPEW; 99.7%; 1.2 kDa) came from GenScript (Nanjing, China).

### 2.2. Bacterial Isolates

In Ruiz-Moyano et al.’s study [14], the acid-tolerance and safety parameters (biogenic amine production and antibiotic resistance) of Serpa autochtonous LAB were studied. Following this, Araújo-Rodrigues et al. [22] studied the technological and protective performance of selected safety autochtonous LAB strains. From this previous study, six LAB strains were selected, namely, *L. paracasei* PC, *L. plantarum* PL1, *L. plantarum* PL2, *Lactobacillus crustorum* CR, *Lactobacillus pentosus* PE and *Levilactobacillus brevis* BR1. These species were isolated from Serpa PDO cheeses after 30 days of ripening [13] and were kept as pure cultures in the bacterial strain collection. The codes in the Ruiz-Moyano et al. study [14] A2LB1, G1Lb5, G2Lb9, A3Lb18, G4Lb18, and C1Lb21 correspond to PC, PL1, PL2, CR, PE, and BR1 in the present study, respectively. The summary of the main technological and protective potential of these strains is presented in Table 1. 

The strains *L. plantarum* PL1 and *L. paracasei* PC were selected as the most promising for starter culture selection, alone or in combination, respectively. The species *L. plantarum* PL2 was also chosen as a control, since this species did not exhibit proteolytic and lipolytic activities in the plate conditions tested. In addition, one strain of the other species under study was also selected, namely, *L. crustorum* CR, *L. pentosus* PE and *L. brevis* BR1, always choosing the strain of each species that showed the best technological and protective performance [22].

### 2.3. Cheese-like Model System

#### 2.3.1. Preparation of Models

Ewes’ milk, produced in the demarked area of Serpa cheese and from the Lacaune sheep breed, aqueous extracts of *C. cardunculus* L. and selected autochthonous LAB, were used to establish the Serpa-cheese model system. The milk was filtered and sterilized at 121 °C for 10 min, and the viable counts in the PC agar medium validated the process. For coagulant preparation, the previously established method of Tavaria et al. [20] was followed. Specifically, 2.5 g of cardoon flowers were macerated in 25 mL of citrate buffer (0.1 M, pH 3.0). The resultant homogenate was centrifuged at 3500 rpm for 10 min and filtered through Whatman No. 1 filter paper. Concerning bacterial inoculation, the conditions tested were: A—*L. paracasei* PC; B—*L. plantarum* PL1; C—*L. paracasei* PC and *L. plantarum* PL1; D—*L. plantarum* PL2; E—*L. crustorum* CR; F—*L. pentosus* PE; G—*L. brevis* BR1; and H—control without inoculation. Condition H was used as a control, without bacterial inoculation. In all cases, the milk used was pasteurized. Bacterial density was determined and adjusted to around 10^8^ colony-forming units (CFU) mL^−1^, the microorganism’s concentration typically found in Serpa PDO cheese. 

A volume of 100 mL of sterilized milk was added to 120 mL sterile flasks and heated at 30 °C. In all conditions established except condition C, 1% (*v*/*v*) of the microorganisms were inoculated in the flasks. In condition C, 0.5% (*v*/*v*) of each bacterial strain was inoculated. Posteriorly, the vegetable extract was added to achieve a final coagulant concentration near the Serpa cheese manufacture (0.8 g L^−1^). The coagulation occurred at 30 °C for 1 h. After this period, the whey was carefully drained without pressing and flasks were incubated at 10 °C until sampling. The sampling points occurred at 0, 15, 30 and 45 days. Analytical duplicates were established for all conditions tested.

#### 2.3.2. Microbiological Analysis and pH Monitorization

At each sampling point, 1 g of curdled milk was mixed in 9 mL of sterile 0.1% peptone solution in a Stomacher Lab-Blender 400 (Seward Medical, UK) for 1 min under aseptic conditions. Serial dilutions were prepared for bacterial density determination. To determine total aerobic bacteria (TAB), the growth was carried out in a PC agar medium after 72 h of incubation at 30 °C [32]. MRS and M17 agar were used to enumerate *Lactobacillus* spp. and *Lactococcus* spp. after anaerobic incubation at 37 °C for 72 h, respectively. Regarding yeasts and molds, the incubation was performed in supplemented RBCA medium for 3 to 5 days at 25 °C, while *Enterobacteriaceae* were enumerated in VRBGA after 24 h at 37 °C. BPA supplemented with egg folk tellurite emulsion was used to grow *Stapylococcus* spp. at 37 °C for 48 h [33]. Microbial count determinations were duplicated, and the results were expressed in the log (CFU g^−1^).

During all sampling points, the pH of the model systems was also monitored using a pH meter (Micro pH 2002, Crison, Barcelona, Spain) in triplicate.

#### 2.3.3. Determination of Proteolytic Indices

Total nitrogen (TN), water-soluble nitrogen (WSN), and soluble nitrogen (SN) in 12% of TCA (TCASN) and 5% of PTA (PTASN), present in cheese models, were measured via the micro-Kjeldahl method, using a Kjeltec system 1002 distilling unit (Tecator; Hoganas, Sweden). To obtain the WSN, the technique of Kuchroo and Fox [34] was followed with a few modifications. An amount of 5 g of each cheese model system were mixed with 15 mL of ultra-pure (UP) water in a Stomacher Lab-Blender 400 for 10 min at 130 rpm. The mixture was incubated for 1 h at 40 °C and centrifuged for 10 min at 4000 min. The sample was then collected in Whatman No. 113 filter paper. 

The TCASN and PTASN were then prepared as described by Tavaria et al. [30]. Briefly, 12% TCASN was obtained by adding 7.5 mL of TCA (48% *w*/*v*) to 22.5 mL of WSN, followed by 30 min of incubation at room temperature and filtration with Whatman No. 42 filter paper. For the 5% PTASN, 20 mL of WSN were mixed with 14 mL of H_2_SO_4_ (3.95 M) and 6 mL of PTA (33.3% *w*/*v*) and incubated at 4 °C overnight. Finally, the mixture was filtered through Whatman No. 542 filter paper. All micro-Kjeldahl determinations were achieved in triplicate.

#### 2.3.4. Protein and Peptide Profile

The protein and peptide profile of the WSN fraction of cheese models was assessed using the AKTA Pure 25 system (GE Healthcare Life Sciences, Freiburg, Germany) by gel size exclusion chromatography (SEC), as described by Voss et al. [35]. This system was coupled with two gel filtration columns: Superdex 200 increase10/300 GL and Superdex peptide, 10/300 GL (GE Healthcare Life Sciences, Freiburg, Germany). The mobile phase used was 25 mM phosphate buffer (pH 7.0), 150 mM sodium chloride and 0.2 g L^−1^ sodium azide, with an eluent flow of 0.5 mL min^−1^. The absorbance was monitored at 280 nm. Some standard proteins were analyzed in the same conditions to establish a molecular weight (MW) standard curve, namely Thyroglobulin (669 kDa), Ferritin (440 kDa), Aldolase (158 kDa), Conalbumin (75 kDa), Ovalbumin (43 kDa), Carbonic anhydrase (29 kDa), Ribonuclease A (13.7 kDa), Aprotinin (6.5 kDa), and an antihypertensive peptide with the sequence KGYGGVSLPEW (1.2 kDa) [36]. UNICORN 7.0 software (Cytiva, USA) was used to analyze the protein and peptide profiles of different cheese models.

#### 2.3.5. Free Amino Acid (FAA) Profile

For FAA analysis, the Pico-Tag™ method [37] was used with some adjustments, as described by Araújo-Rodrigues et al. [31]. In the first phase, deproteinization and FFA extraction were carried out, homogenizing each cheese model with perchloric acid (0.6 N; 1:10) in an Ultra-turrax^®^ (T18, IKA) for 2 min at 12,000 rpm. The homogenate was then centrifuged (20 min; 3500 rpm) and filtrated with Whatman No. 1 paper. The sample pH was adjusted to 7.1 ± 0.2 and incubated on ice for 5 min. After incubation, the extract was filtered with a 0.45 µm filter. The high-performance liquid chromatography (HPLC) analysis was used according to the Pripi-Nicolau et al. method [38]. The amino acid standards (18 standards) were prepared, and through a comparison of their retention time and regression curves, FAA in cheese models were identified and quantified. Homoserine and norvaline were used as internal standards. For each cheese model, FAA determination was performed in duplicate.

#### 2.3.6. Volatiles Fatty Acids (VFA) Profile

Headspace solid-phase microextraction (HS-SPME) coupled with gas chromatography and mass spectrometry (GC–MS) was used to determine the VFA present in cheese models according to the conditions described by Araújo-Rodrigues et al. [31]. For the experiment, flasks of 20 mL (capped with a gastight seal) were used, adding 1 g of each cheese-like model and 10 µL of internal standard (50 mg L^−1^ of 3-octanol). A Divinylbenzene/carboxen/polydimethylsiloxane (DVR/CAR/PDMS; Supelco Co., Bellefonte, PA, USA) was used for volatile adsorption for 1 h at 60 °C. The SPME fiber was introduced into the injector port for volatile desorption and left to desorb the trapped volatiles for 15 min. A Varian CP-450 gas-chromatograph (Walnut Creek, CA, USA) with an SGE GC column BP21 (FFAP; 50 m × 0.22 mm × 0.25 µm) from BGB Analytik (Böckten, Switzerland) was used. Helium was used as carrier gas under a 1.0 mL min^−1^ constant flow. The mass spectra were acquired in electron impact (EI) ionization mode at 70 eV. The ion source temperature was 210 °C, while the transfer line temperature was 160 °C. To scan the mass spectra and acquire data, the 33–350 m/z range and Varian Saturn 240 MS were used. Standard curves of VFAs were prepared under the same conditions, and their retention time was used for identification, coupled with the mass spectra obtained compared with the mass spectra of the NIST 98 MS library database.

### 2.4. Statistical Analysis

In order to evaluate the significant differences between the different LAB strains tested, the one-way analysis of variance (ANOVA) was used, recurring to SPSS Statistics 28.0.1 (IBM, Armonk, NY, USA), at a degree of significance of *p* < 0.05. The normal data distribution was confirmed, and the ANOVA was used to compare the experimental conditions statistically. Post-hoc multiple comparisons were carried out using the Tukey’s test (*p* < 0.05).

Principal Component Analysis (PCA) was used to compare the performance of the LAB inoculation in model cheeses based on all variables evaluated (STATISTICA 8.0 package, StatSoft, Tulsa, OK, USA). Successive analyses were performed using all variables. A set of biochemical markers that were sufficiently discriminative regarding LAB strain action were selected, providing a greater explained total variance by the first three principal components (PC). 

To compare the technological performance of LAB strains in cheese models with Serpa cheese, four PDO cheeses were simultaneously analyzed. Several sensory, physical, and chemical parameters of this cheese sample were determined by Araújo-Rodrigues [31]. The Serpa cheeses were manufactured according to the PDO specifications and produced for four consecutive months (two in Winter and two in Spring). The samples were coded as DEF, AEM, AEA, and AEMy, and the total score obtained by a qualified accredited panel was 16.6, 17.9, 17.2, and 17.4, respectively. Regarding the taste and smell parameter, the score was approximately 4.8 to DEF, 5.1 to AEM, 5.0 to AEA, and 4.9 to AEMy [31]. Accordingly, all cheeses were classified as “Excellent” and accepted for the PDO certification [4,10]. The same PCA approach was used to compare the performance of LAB inoculated in cheese models with microorganisms present in the PDO cheeses based on all variables evaluated.

## 3. Results and Discussion

Cheese models or miniature cheeses have been used in recent decades to prospectively study the effect of several technological factors on cheese properties [20,24,25,26,27]. In the present study, different cheese models were established (described as conditions A, B, C, D, E, F, G, and H) to assess the potential in the cheese environment of the selected autochthonous bacterial strains of Serpa cheese. During the different sampling points (0, 7, 15, 30, and 45 days), the pH, main microbial groups, and key chemical markers involved in the organoleptic properties (volatiles, peptides, and FAAs), and soluble nitrogen fractions (PTASN and TCASN) were monitored in the cheese models.

### 3.1. Microbiological and pH Monitorization

During the cheese model ripening, the main microbiological groups present in dairy products were monitored. The sterilization of ovine milk used for cheese model preparation was validated, and no microbial counts were detected. In all conditions and maturation times, microbial counts were not detected in the BPA medium. An increase in TAB was registered in all conditions, with no key variation from 30 days of ripening. Although no microorganism was inoculated in condition H (control), the natural microbial load of cardoon contributes to the microbiological counts and can be seen as a microflora naturally present in Serpa cheese, being transversal to all conditions. After 45 days of ripening, the TAB counts were approximately 4.42 log (CFU g^−1^). Aquilanti et al. described the microbiological profile of cardoon [39], indicating the presence of, for instance, *Lactobacillus* spp., *Lactococcus* spp., and coliforms. After 45 days of ripening in conditions A, B, C, D, E, F, and G, the TAB suggested counts between 8.11 and 9.54 log CFU g^−1^. These results were close to the natural microbial load of Serpa PDO cheese, typically between 8 and 9 log CFU g^−1^ [13,14]. During all experimental points, the main microbial group were *Lactobacillus* spp., while *Lactococcus* spp., yeasts, molds, and Enterobacteriaceae minority groups resulted from natural cardoon microflora. 

Regarding the pH assessment over 45 days, significant differences between the cheese models with different LAB strains inoculated during ripening time were registered, and the results are described in Table 2. As expected, in condition H, where no strain was inoculated, only a slight decrease in pH over the 45 days was verified, suggesting significant differences between this group and the other conditions in all sampling times. The control exhibited a higher pH value after 45 days of incubation, approximately 5.36. This slight acidification may result from some acidifying activity of the LAB and other bacteria in the cardoon’s natural microflora [39]. Additionally, the cardoon action may be directly involved in this acidification by releasing peptides from casein, as suggested by the pH evolution in model H, corresponding to heated milk with the addition of a coagulant. As suggested by Tavaria et al., a decrease in pH was verified in plant-coagulated cheeses, while in cheeses manufactured using animal rennet, a slight increase was registered [20].

After 45 days of ripening, all cheese models showed a pH value below 5, except condition G (inoculated with BR1). According to the results presented in Table 2, the strain PE showed greater acidifying capacity, while the BR1 showed the lowest acidifying potential. Although PC showed a lower acidification ability compared with the other strains (PL1, PL2, CR, and PE) in skim milk medium [22] and in the cheese model microenvironment (Table 2), its combination with PL1 (condition C) resulted in a significant higher decrease in pH (approximately 4.31 alternatively to 4.62). In a study focused on Serra da Estrela PDO cheese, a decrease in pH from 6.5 to 4.8 in cheese models was reported. Regarding Serpa PDO cheese, a recent study that focused on sensorial and physicochemical parameters suggested a pH of approximately 4.98 for cheeses classified as “excellent” in the sensorial analysis, with at least 30 days of ripening [31]. 

The cheese models were a simplified microenvironment, dominated mainly by the inoculated LAB, lacking the presence of several key microorganisms such as yeasts, molds, and nonstarter LAB (NSLAB). During the first stage of cheese production, cheese microbiota is dominated by starter LAB (SLAB), responsible for lactose fermentation and lactic acid production [4,13]. However, during the ripening period, the cheese microbiota is dominated by NSLAB, which can resist hostile conditions (deficiency of nutrients, high salt concentration, low temperature, pH, and moisture). Yeasts also play an essential role in the cheese environment through ripening [40]. Both groups possess several hydrolytic enzymes that contribute to the final organoleptic profile. For instance, their enzymes promote a pH increase, which is important in texture development [41]. The absence or minority presence of these microorganisms in the cheese models avoided the natural neutralization that occurs in the cheese environment in the final ripening phase.

### 3.2. Proteolysis

#### 3.2.1. Protein and Peptide Profile

**Figure 1:**
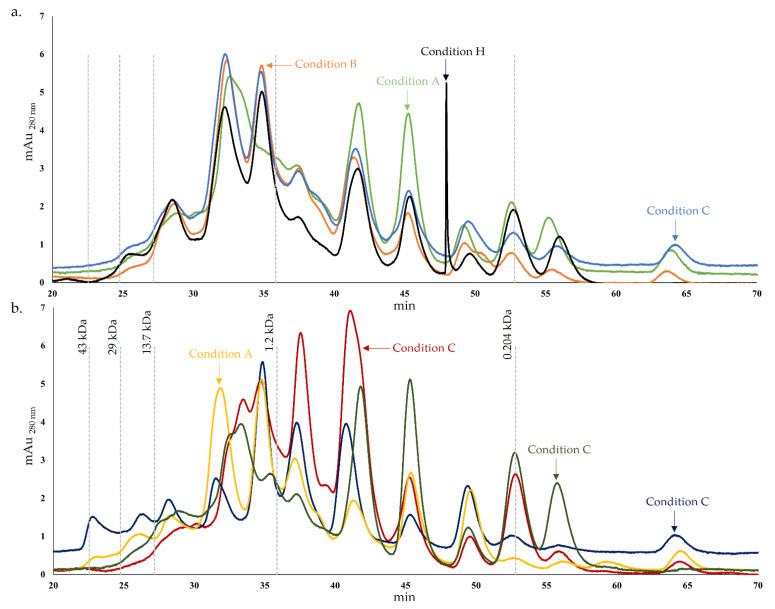
Protein and peptide chromatogram of cheese models after 45 days of ripening. (**a**). – Condition A (*L. paracasei* PC), – Condition B (*L. plantarum* PL1), – Condition C (*L. paracasei* PC and *L. plantarum* PL1), – Condition H (without inoculation); (**b**). – Condition D (*L. plantarum* PL2); – Condition E (*L. crustorum* CR); – Condition F (*L. pentosus* PE) and – Condition G (*L. brevis* BR1).

Proteolysis has been considered the essential biochemical event for flavor and texture development during ripening [30,42]. The protein and peptide chromatograms of cheese models in the final ripening time are represented in Figure 1. Generally, the protein and peptide profile was similar in all conditions, with a higher concentration of peptides with an MW lower than 13.7 kDa and suggesting a high diversity of peptides. Throughout cheese production and ripening, proteins are sequentially degraded by primary and secondary proteolysis, resulting in polypeptides and, subsequently, in medium and small-size peptides and FAA [30,43]. Regarding primary proteolysis in cheese, it has been associated with the softening of texture since this event promotes three-dimensional protein matrix disruption [42]. The primary proteolytic action results mainly from C. cardunculus L. action, so the protein and peptide profile is similar in all conditions. The cardoon extract possesses several proteinases, such as cardosins, cyprosins, and cynarase, analogous to other aspartic proteinases [44,45,46]. These enzymes hydrolyze specific casein bounds, promoting milk coagulation and producing a casein gel [44]. Cardoon proteinases are reported as highly proteolytic and also have intense secondary proteolytic action against α_S_- and β-caseins [44,45,46,47].

However, the peptide concentrations were different in the conditions analyzed. The peptides resulting from secondary proteolysis influence the cheese flavor and are targets of proteinases and peptidases from cheese microflora [42]. In terms of peptide concentration, the chromatogram of condition C is very similar to condition B until approximately 45 min. For lower MW, the concentration of peptides from condition C is typically more similar to condition A or, in some cases, the chromatogram suggested intermediate concentrations of peptides between condition A and B (Figure 1a). This may be explained by condition C, which corresponds to the cheese model resulting from the inoculation of the strains PC and PL1 mixture present in conditions A and B, respectively. Consequently, the proteases of these strains possess different proteolytic specificities, resulting in a combined pattern. Generally, the results suggested that condition A exhibits a higher concentration of peptides lower than 1.2 kDa compared with condition B and C, which may indicate a higher proteolytic action. Comparing these chromatograms (A, B and C) with condition H, typically, the control exhibit similar or lower peptide concentrations. A distinctive peak was registered in condition H (around 48 min), which probably resulted from a peptide hydrolyzed in the other conditions by proteases from *Lactobacillus* spp.

**Figure 2:**
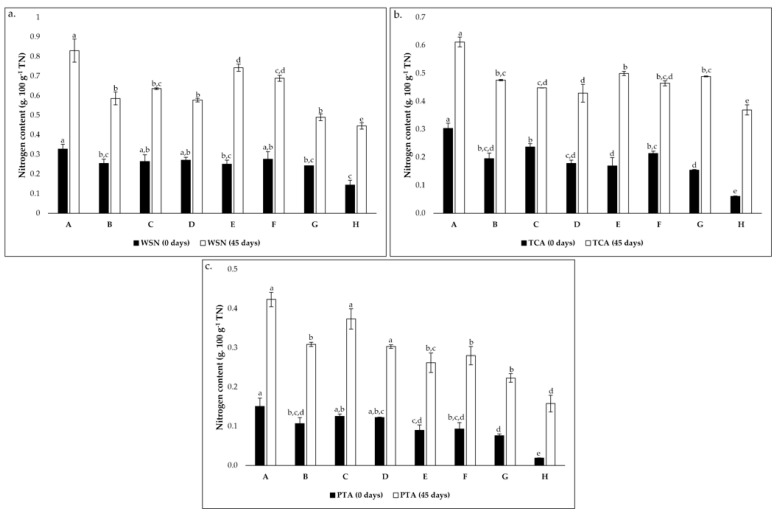
Evolution of nitrogen content (g 100 g^−1^ total nitrogen) from 0 to 45 days of incubation in cheese models (**a**). for water-soluble nitrogen (WSN); (**b**). TCA-soluble nitrogen (TCA) and (**c**). PTA-soluble nitrogen (PTA). Conditions A—*L. paracasei* PC; B—*L. plantarum* PL1; C—*L. paracasei* PC and *L. plantarum* PL1; D—*L. plantarum* PL2; E—*L. crustorum* CR; F—*L. pentosus* PE; G—*L. brevis* BR1; and H—without inoculation. In each graph, different superscript letters on bars of the same color indicate significant differences between conditions (*p* < 0.05).

Regarding Figure 2b, as previously mentioned, although a typical pattern is normally registered in all chromatograms, the peptide concentrations were distinct in the different conditions. The chromatogram of condition D stood out from the others by a higher concentration of peaks corresponding to higher MW peptides, with MW between approximately 1.5 and 0.8 kDa. For lower MW, a lower concentration of peptides was registered for condition D, which may indicate a lower proteolytic potential. This theory may align with the results of a previous study where the absence of extracellular proteolytic activity in PC agar medium supplemented with skim milk was reported for the PL2 strain [22]. On the other hand, this species may possess proteolytic activity in the cheese environment but with a more distinctive pattern than the other *Lactobacillus* spp. strains used, which may be clarified with the analysis discussed below. The pattern of condition G showed some incidence in lower MW peptides, while conditions E and F showed a greater preponderance for the production of peptides with lower or intermediate MW.

#### 3.2.2. Soluble Nitrogen Assays

The evolution of soluble nitrogen fractions, namely WSN, TCASN, and PTASN are recognized as key indicators of proteolysis in cheese [48]. These parameters are presented in Figure 2. The WSN possesses a rich and distinct composition of whey proteins and peptides with diverse MW and FAAs [20]. Throughout ripening, an increase of WSN is expected due to the primary action of cardoon proteinases on milk casein [8,43,48]. As suggested in Figure 2a, there is an increase in the coefficient between WSN and TN during the 45 days of ripening in all conditions. However, there are significant variations of nitrogen content according to the condition, mainly after 45 days of ripening. In the initial ripening phase, this coefficient varied from approximately 0.14 to 0.33 g 100 g^−1^ TN while, after 45 days, it varied from approximately 0.45 to 0.83 g 100 g^−1^ TN. The lower and upper values correspond to the control condition (condition H) and condition A, respectively. These were transversal to TCASN (Figure 2b) and PTASN (Figure 2c), as well as the increasing tendency during ripening time, although this tendency was higher in these cases than in the WSN. This suggests a strong effect of the LAB inoculation in the cheese models.

The evolution of TCASN can be seen as a “ripening depth” index since, at the concentration of 12% TCA, most WSN peptides and FAA precipitate [30,49]. The small peptides and FAA precipitated at these conditions are mainly a result of the proteolytic action of starter microorganisms [43,48,49]. In addition, an increase in TCASN was registered (Figure 2b) as expected [30,49], and significant variations were verified. The values ranged between around 0.06 and 0.30 g 100 g^−1^ TN in the initial phase and between approximately 0.37 (condition H) and 0.61 g 100 g^−1^ TN (Condition A) in the final ripening time.

Finally, 5% of PTASN precipitates molecules <600 Da, including mainly FAA but also very small peptides [49]. The same increasing tendency of WSN and TCASN was registered for PTASN, and significant variations were verified between conditions (Figure 2c). After cheese model production, the coefficient between PTA and TN varied between around 0.02 and 0.16 g 100 g^−1^ TN, corresponding to conditions H and A, respectively. Regarding the final ripening time, the coefficient ranged from approximately 0.16 (condition H) to 0.42 g 100 g^−1^ TN (condition A).

In all cases, condition A showed the highest nitrogen content, which may indicate a higher proteolytic action of strain PC. This study highlights the role of LAB in proteolysis, mainly in the depth of proteolysis. All values were lower than those reported for Serpa PDO cheese [31,49,50] and other raw milk ovine cheeses [30,51], since the present study’s cheese models were used. As previously mentioned, the absence or minority presence of other microbial groups (NSLAB and yeasts) in the cheese models, which possess a crucial role in cheese ripening, strongly limits the cheesemaking process.

#### 3.2.3. Free Amino Acids (FAA) Content

FAA play key roles in the typical cheese aroma and flavor. Each cheese is associated with a distinctive FAA profile, which results from the presence of particular enzymes and specific degradation and inter-conversion mechanisms [45]. As expected, an increase in FAA concentration was registered for all conditions established. The FAA content of cheese models after 45 days of ripening is present in Table 3. Significant differences in each FAA and high diversity of FAA were registered in the conditions tested. Given these variations, the most incident FAA in all conditions (except control—condition H) was Leu, with concentrations ranging between approximately 121.31 and 362.85 mg 100 g^−1^. Each cheese model possessed a specific FAA profile, which corroborated the importance of specific cheese microflora in the particular sensorial profile of each cheese type [45].

Beyond Leu, in the cheese model corresponding to condition A, Glu, Ala, Tyr, Val, and Phe were the most prevalent groups, while in condition B they were Arg, Ala, Tyr, and Phe. Concerning condition C, which combines the strains PC and PL1 (in a single form in conditions A and B, respectively), following Leu, the higher concentrated FAA were Glu, Ala, Arg, Tyr, Val, and Phe. This shared FAA profile probably resulted from the combined proteolytic action of both strains. Arg, Ala, Tyr, Val, and Phe were between the most incident FAA in condition D, and Arg, Ala, Val, and Phe in condition E. Condition F showed that beyond Leu, Glu, Phe and Val possessed concentrations higher than approximately 65.87 mg 100 g^−1^ and, in condition G, Phe, Val and Tyr presented concentrations higher than approximately 53.48 mg 100 g^−1^. Regarding the control condition, only Asp, Glu, Ser, Ala, Tyr, Val, Met, and Ile were detected in concentrations ranging between approximately 5.18 and 78.55 mg 100 g^−1^. Glu, Ala, Tyr, and Ile were the most prevalent in this case. In a previous study, the characteristic FAA pattern of Serpa PDO cheese was determined. Asp, Glu, Ala, Leu, Val, and Phe were reported as the most prevalent FAA, with concentrations ranging between 18.76 and 1445.16 mg 100 g^−1^ [31]. Leu, Val and Phe are typical incident FAA in ewe, cow, goat, and mixed cheeses [30,52,53].

As expected, the control condition exhibited a significantly lower concentration of FAA since no strain was inoculated in this cheese model. Cardosins, cyprosins, and cynarase from vegetable coagulants and proteolytic enzymes from residual microflora present in vegetable coagulants have reduced FAA production. The hypothesis that strain PC (condition A) exhibited higher proteolytic action by the analysis of protein and peptide chromatogram (Figure 1) and soluble nitrogen assays (Figure 2) may be corroborated by the significantly higher content of total FAA in this condition (ca. 1460.57 mg 100 g^−1^). Alternatively, the hypothesis that PL2 did not exhibit extracellular proteolytic activity may not be supported by the FAA results, since the results suggested a high concentration of total FAA, around 1111.11 mg 100 g^−1^. In the plate medium tests, the proteolytic effect is based on the milk protein degradation that is part of the culture medium [54]. Alternatively, in the cheese model, the microbial effect is fundamentally exerted on the casein modified by the action of the coagulant [55].

Although the cheese model inoculated with strain PL1 exhibited a lower concentration (ca. 843.51 mg 100 g^−1^) compared with condition A and D cheese models, combined with strain PC (condition C) resulted in a higher concentration of FAA, around 1218.54 mg 100 g^−1^. Regarding conditions E, F, and D, the results suggested significantly lower concentrations of total FAA, ranging from approximately 626.78 to 718.02 mg 100 g^−1^. This may be the result of the lower proteolytic action of CR, PE, and BR1 strains enzymes or a more specific proteolytic action, which hydrolyses fewer amino acid residues of casein peptides resulting from vegetable coagulant hydrolysis. On the other hand, higher concentrations of FAA may indicate their lower conversion into volatile molecules [20].

Regarding the total identified and quantified FAA in Serpa PDO cheese, in the cheeses sensorial classified as “excellent”, the values varied from ca. 2374.81 to 3804.93 mg 100 g^−1^ [31]. This fact may raise the hypothesis that a high concentration of FAA plays an essential role in Serpa PDO cheese specificity. Strains such as PC, PL2, and a combination between PC and PL1, which result in cheese models with a higher concentration of total FAA, could be favorable to an autochthonous starter culture since it may promote the development of organoleptic properties closer to this traditional product.

### 3.3. Volatiles

#### 3.3.1. Volatile Fatty Acids (VFA)

Numerous volatiles are generated during the complexity of the ripening process, with an increase reported in these molecules throughout this phase [56]. Accordingly, the results of this study suggested an increase in volatiles, namely in VFA and esters, during ripening. *C. cardunculus L.* and other vegetable rennets play essential roles in the production of volatiles. They are responsible for the higher digestibility, more intense flavor, and aroma of vegetable-coagulated cheeses [57]. In the cheese environment, the degradation of lipids by lipase/esterase enzymes is the main contributor to fatty acids (C_4_ to C_18_) production [58]. However, the degradation of FAA and sugars (principally lactose) also results in the generation of butyric acid (C_4_) and C_6_ [29]. Milk and its microorganisms are the primary sources of lipases. However, during milk pasteurization, the milk enzymes are deactivated by the effect of temperature [59].

Table 4 summarizes the results of VFA in cheese models after 45 days of ripening. The most incident VFA were C_6_, C_8_, C_10,_ and C_12_. These results align with Araújo-Rodrigues et al.’s [31] study, where the volatile profile of Serpa PDO cheese was studied. Specifically, C_6_, C_8,_ and C_10_ were the most prevalent VFAs in all cases, except in condition G, where C_12_ showed a higher concentration than C_6_. Beyond Serpa, C_6_ is also reported as one of the most prevalent VFA in other ewes’ raw milk cheeses [20,57,59]. This molecule is associated with aromas such as sweety, goaty, soapy, and waxy, resulting from the catabolism of Val [59,60]. C_8_ is typically more concentrated in ovine than in cow milk. Soapy flavors are typically associated with C_10_ and C_12_ molecules [61]. C_10_ was the most common VFA in conditions A, B, D, and G (with concentrations varied around 203.42 and 882.07 mg 100 g^−1^), C_6_ in conditions C and E (ranging between approximately 712.74 and 1689.07 mg 100 g^−1^), and C_8_ in conditions F (with values around 910.45 mg 100 g^−1^). Concerning the control condition, the concentrations of the three most prevalent VFAs varied from 16.56 and 26.09 mg 100 g^−1^. In this condition, iC_4_, C_5_, C_7_, C_9,_ and C_12_ showed values between 0.31 and 2.72 mg 100 g^−1^.

Regarding total VFA, the control condition exhibited a significantly lower concentration (ca. 69.86 mg 100 g^−1^), aligning with Tavaria et al.’s study [20]. In this study, the authors reported that the cheese model containing only the coagulant showed a considerably lower amount of volatiles. This demonstrates that the activity of microorganism enzymes on hydrolyzed proteins is the key factor for volatile production and the minority presence of lipolytic enzymes in cardoon [20]. The results suggested significant variations in total VFA in distinct cheese models. Condition E exhibited higher total VFA concentrations, approximately 3949.74 mg 100 g^−1^. Excluding condition H (control), this condition demonstrated the lower FAA concentration (Table 3), which was probably related to the higher conversion of FAA to volatiles [20]. The same trend occurred in condition F, which was also one of the conditions that exhibited a lower concentration of FAA but high levels of VFA. On the contrary, a lower concentration of VFA was also quantified beyond the low FAA registered in condition G. A lower concentration of VFA (except the control) was verified for this condition, specifically 380.65 mg 100 g^−1^. Despite the apparent low conversion of FAAs in VFA in condition G, the conversion to other volatiles may occur, for example, in esters. At the same time, sugars, namely lactose, are also degraded, contributing to volatile production, such as C_4_ and C_6_. Regarding conditions A, C, and D, the total VFA quantified were closer, ranging between approximately 2151.28 and 2481.52 mg 100 g^−1^. However, higher concentrations of FAA were also registered (Table 3). In accordance with FAA results, condition B showed a lower total VFA than conditions A, C, and D. In Serpa PDO cheese, a higher range of total VFA concentration was identified, between 323.21 and 3986.03 mg 100 g^−1^ [31]. This may corroborate the high variability of these traditional products and the high impact of microflora in volatile formation.

#### 3.3.2. Esters

With regard to esters, the esterification between alcohols and carboxylic acids, as well as alcoholysis between acylglycerols and alcohols, are key reactions involved in the biosynthesis of esters [62]. These molecules are involved in the final product flavor, namely in the fruity annotations and in the reduction in sharpness and bitterness. Typically, some esters have a low perception threshold [58,63,64]. Esters identified and quantified in the conditions under study are present in Table 5.

Ethyl octanoate, decanoate, and dodecanoate were the most prevalent esters in all conditions, with concentrations ranging between approximately 15.16 and 445.79; 24.61 and 1954.83; and 22.33 and 878.09 mg 100 g^−1^, respectively. The key role of ethyl octanoate in cheese aroma formation has been reported [65]. The ester compounds in cheese play an important role in forming sweet, fruity, and floral aromas. However, excessive ethyl butyrate and ethyl caproate lead to an overpowering fruity flavor defect [66]. In condition D, ethyl butanoate (around 23.27 mg 100 g^−1^) and hexanoate (approximately 23.13 mg 100 g^−1^) were also among the most concentrated esters. Ethyl butanoate has been described as a strong odorant in different types of cheeses, while the significant role of ethyl hexanoate in cheese aroma has also been reported [63]. All esters detected and quantified showed a significantly higher concentration in condition G. Ethyl isovalerate was only detected in condition G, while ethyl acetate was not detected in the conditions studied. No significant variations were found between the other conditions in the case of ethyl valerate, nonanoate, decanoate, and dodecanoate. Ethyl isovalerate, ethyl heptanoate, and ethyl nonanoate were detected in almost all Serpa cheeses analyzed by Araújo-Rodrigues et al. [31]. In contrast, ethyl butyrate, ethyl hexanoate, ethyl octanoate, ethyl decanoate, and ethyl dodecanoate reached higher average values for detected esters.

Significant variations were also registered in the ester concentration present in each condition and total ester values. As registered in the total FAA and VFA results, the control condition also showed a lower concentration of total esters, which was discussed previously. The higher values of total esters were registered for condition G, which aligns with the hypothesis that low FAA may result from the high conversion in volatiles [20], in this case in esters. No significant differences were verified between total VFA in conditions A and E, with values around 384.32 and 421.37 mg 100 g^−1^. Significant lower values (approximately 222.83 mg 100 g^−1^) were registered to condition F. Conditions C, B, and D exhibit lower total ester values (except control), varying from 98.43 and 117.15 mg 100 g^−1^.

In a previous study, the total identified and quantified esters in Serpa PDO cheeses, classified as “excellent” in the sensorial analysis, were between 1.23 and 28.7 mg 100 g^−1^. In these cheeses, ethyl acetate, ethyl butanoate, and hexanoate were not detected [31]. In all cheese models, the total ester concentrations were higher than in Serpa PDO cheese.

### 3.4. Results Integration

**Figure 3:**
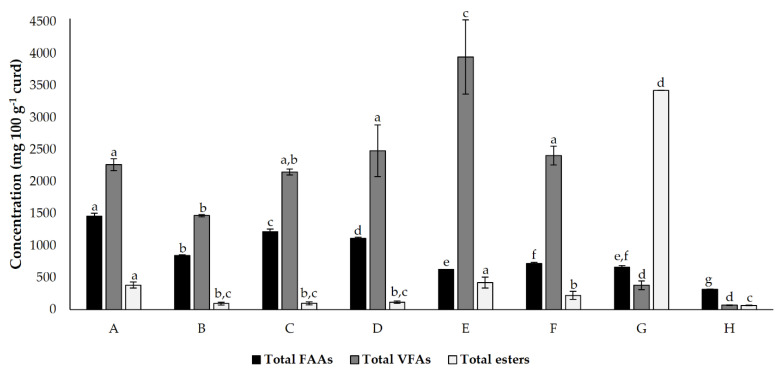
Total detected and quantified (mean ± standard deviation) free amino acids (FAA), volatile fatty acids (VFA) and esters present in cheese models (mg 100 g^−1^), incubated for 45 d. Different superscript letters in the same bar colour indicate significant differences between conditions (*p* < 0.05). Conditions A—*L. paracasei* PC; B—*L. plantarum* PL1; C—*L. paracasei* PC and *L. plantarum* PL1; D—*L. plantarum* PL2; E—*L. crustorum* CR; F—*L. pentosus* PE; G—*L. brevis* BR1; and H—without inoculation.

The total FAA, VFA, and esters in each cheese model are summarized in Figure 3. The different cheese models tested exhibited significant differences between the total FAA, VFA, and esters. As expected, the control condition showed a lower total FAA, VFA, and ester concentration. In this condition, the presence of these molecules is limited to their natural existence in milk and is generated by microbial action (which only results from natural cardoon microbiota). In this graph, the fact that a lower accumulation of FAA may result in a higher conversion of volatiles is more evident for condition E (where the higher concentration of VFA was registered), condition F, and condition G (where the higher prevalence of esters was registered).

**Figure 4:**
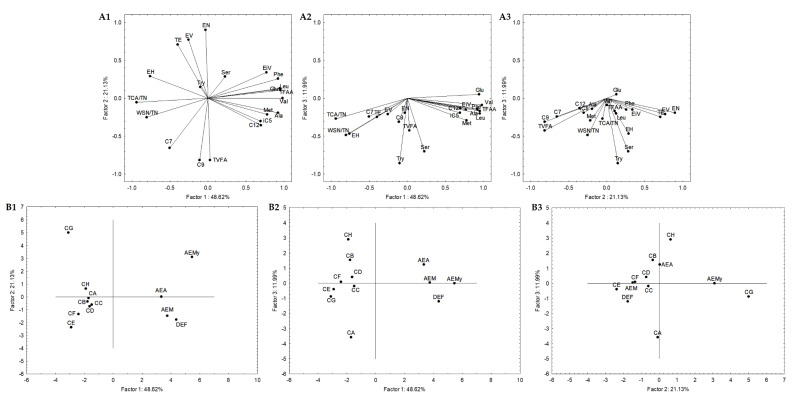
Variable projection (A) and samples projection (B) of principal component analysis. Variable projection of PC1 vs. PC2 (**A1**), PC1 vs. PC3 (**A2**) and PC2 vs. PC3 (**A3**). Sample projection of PC1 vs. PC2 (**B1**), PC1 vs. PC3 (**B2**) and PC2 vs. PC3 (**B3**). DEF, AEM, AEA and AEMy correspond to Serpa PDO cheeses and conditions CA—*L. paracasei* PC; CB—*L. plantarum* PL1; CC—*L. paracasei* PC and *L. plantarum* PL1; D—*L. plantarum* PL2; E—*L. crustorum* CR; F—*L. pentosus* PE; G—*L. brevis* BR1; and H—without inoculation.

In order to select the most promising autochthonous LAB from Serpa PDO cheese to develop a starter culture to test in a natural cheese environment, subsequent statistical analyses were carried out to reduce the number of variables. The attributes that did not significantly impact the analysis were excluded, increasing the explained variance. Four Serpa PDO cheeses produced in consecutive months were included and compared with cheese models in the first adopted approach. Accordingly, 21 attributes (variables) were selected. These attributes were: the quotient between WSN and TN and TCA and TN; the amino acids Glu, Ser, Ala, Val, Met, Tyr, Leu, Phe and total FAA; the VFA iC_5_, C_7_, C_9_, C_10_, and total VFA; the esters ethyl isovalerate, valerate, hexanoate and octanoate, and total esters. The variables projection in the three planes constituted by the three principal components (PC) is shown in Figure 4. Together, these three PC (PC1, PC2, and PC3) explained 81.74% of the total variance. PC1, PC2, and PC3 condensed 48.62%, 21.13%, and 11.99% of the total variance, respectively. The similarity map defined by PC1 and PC2 accounted for 69.75% (Figure 4(A1)) of the total variance, PC1 and PC3 with 60.61% (Figure 4(A2)), and PC2 and PC3 with 33.12% (Figure 4(A3)).

The PC1 presented negative correlations with ethyl hexanoate (r = −0.76), quotients of WSN and TN (r = −0.81) and TCA and TN (r = −0.94), and positive correlations with Glu (r = 0.94), Ala (r = 0.92), Val (r = 0.98), Met (r = 0.78), Phe (r = 0.92), Leu (r = 0.95), Total FAA (r = 0.96) and ethyl isovalerate (r = 0.77) (Figure 4(A1,4A2)). It is clearly a component related to proteolysis. Concerning PC2, it presented negative correlations with C_9_ (r = −0.82), total VFA (r = −0.81) and positive correlations with ethyl valerate (r = 0.77), nonanoate (r = 0.90) and total esters (r = 0.71) (Figure 4(A1,4A3)), clearly a component related with secondary microbial metabolism. Finally, PC3 is negatively correlated with Ser (r = −0.70), and Try (r = −0.85) (Figure 4(A2,A3)), expressing in a way the overall depth of proteolysis. This may suggest that proteolysis indicators mainly characterize PC1, while PC2 is from compounds resulting from secondary metabolisms such as VFA and esters.

Generally, in the sample projections, the cheese samples are grouped (PC1 vs. PC2 and PC1 vs. PC3, Figure 4(B1,B2)) but on the opposite side of the cheese models’ plane. The present cheese models consisted of a simplified prototype with a limited microbiota. Thus, they were used to comprehend the effect of inoculated Serpa autochthonous LAB on proteolysis and lipolysis. However, cheeses and cheese models are very different microenvironments, which may justify their opposite plan projection (Figure 4(B1,B2)). The differences are mainly related to the distinct concentrations of the molecules in cheese and cheese models. Regarding PC2 vs. PC3, the cheese samples DEF, AEM and AEA are aggregated in the same plane or closer to conditions B, C and D. Although the variations between the two groups of samples were analyzed, this may suggest a higher proximity of these cheese models to the Serpa PDO cheese in terms of lipolytic and proteolytic events. In conditions B, D, and C, strains from the *L. plantarum* species were inoculated (in the case of condition C, a mix). These results may agree with several works showing the presence and importance of *L. plantarum*, considered NSLAB, in defining the properties of some traditional cheeses, including Portuguese cheeses [41,57]. In this projection, the cheese sample AEMy was on an opposite plane to the others, representing the significant variability of traditional cheeses produced with raw ovine milk.

**Figure 5:**
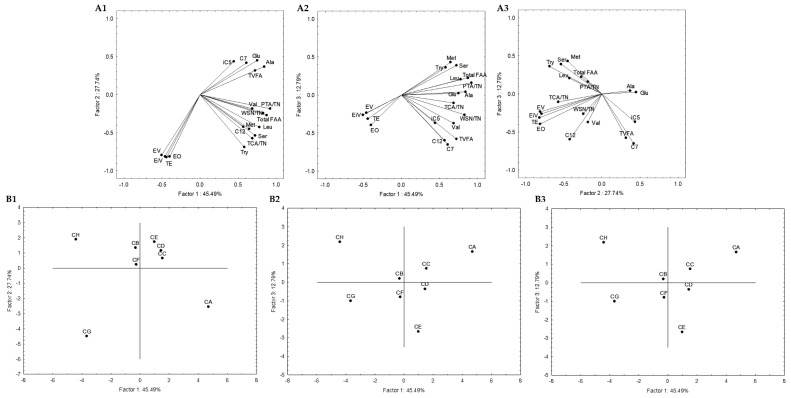
Variable projection (A) and samples projection (B) of principal component analysis. Variable projection of PC1 vs. PC2 (**A1**), PC1 vs. PC3 (**A2**) and PC2 vs. PC3 (**A3**). Sample projection of PC1 vs. PC2 (**B1**), PC1 vs. PC3 (**B2**) and PC2 vs. PC3 (**B3**). CA—*L. paracasei* PC; CB—*L. plantarum* PL1; CC—*L. paracasei* PC and *L. plantarum* PL1; D—*L. plantarum* PL2; E—*L. crustorum* CR; F—*L. pentosus* PE; G—*L. brevis* BR1; and H—without inoculation.

Secondly, a statistical analysis including only cheese models was carried out. Subsequent statistical analyses were also performed, and 19 attributes were selected. These attributes were: the quotient between WSN and TN, PTA and TN, and TCA and TN; the amino acids Glu, Ser, Ala, Val, Met, Tyr, Leu, and total FAA; the VFAs iC_5_, C_7_, C_12_, and total VFAs; the esters ethyl iso-valerate, valerate and octanoate, and total esters. Figure 5 represents the variables projection in the three planes, which are PC1, PC2, and PC3. PC1 condensed itself 45.49%, PC2 27.74%, and PC3 12.79%. These three planes explained 86.02% of the total variance, while PC1 and PC2 explained 73.23%, PC1 and PC3 explained 58.28%, and PC2 and PC3 accounted for 40.53%. PC1 showed positive correlations with quotients between WSN and TN (r = 0.83) and PTA and TN (r = 0.92), Glu (r = 0.75), Ser (r = 0.73), Ala (r = 0.84), Leu (r = 0.78), total FAA (r = 0.87), and total VFA (r = 0.73). PC2 presented negative correlations with ethyl isovalerate (r = −0.79), valerate (r = −0.81) and octanoate (r = −0.81) and total esters (r = −0.82). We again found PC1 representing proteolysis (Figure 5(A1,A2)), while PC2 may be considered as the esters component, with a small contribution of the VFA (Figure 5(A1,A3)).

Generally, conditions B, C, D and F are aggregated in all sample projections (Figure 5(A1–A3)). This may indicate that the strains belonging to *L. plantarum* species or the mixed condition (PL1 and PC strains) are closer in terms of lipolytic and proteolytic events to *L. pentosus* PE. These were also aggregated with condition E in the similarity map defined by PC1 and PC2. These results aligned with the sample projections presented in Figure 4, where conditions B, C and D were aggregated, allowing the consolidation of the information provided by the first PCA analysis described.

## 4. Conclusions

The cheese microbiota plays a fundamental role in organoleptic profile development. In Serpa PDO cheese legislation, pasteurization and starter culture inoculation are prohibited. Thus, in traditional raw milk cheeses, the microbiota is of particular relevance since, beyond the processing technology, their typical sensorial properties are strongly dependent on several parameters (e.g., feedstock, milking, and environmental conditions). These factors greatly influence the complex microbiota present in raw milk, suggesting a significant heterogeneity. Over the years, traditional cheesemaking has incorporated modifications (e.g., controlled ripening, refrigeration) that also impact the microbiological profile of raw milk. Consequently, uncertainty regarding food safety and ripening evolution results in flavor, texture, and safety shortcomings. An effective strategy to minimize or overcome these issues is the development of an autochthonous starter culture.

One of the major challenges in developing an autochthonous starter culture corresponds to the proper selection of microorganisms. In the present work, some autochthonous Serpa PDO cheese microorganisms previously selected based on their safety, technological and protective performance were tested in laboratory-scale cheeses. The impact of these strains on acidification, proteolysis (protein and peptide profile, nitrogen fractions, FAA), and volatile generation (VFA and esters) was investigated. The impact of different strains was evident, with significant differences being found in all parameters investigated. The control condition (without LAB inoculation) showed a lower total FAA, VFA, and esters concentration. In this condition, the presence of these molecules is limited to their natural existence in milk and microbial action (only resulting from natural cardoon microbiota). In some conditions established, a lower accumulation of FAA resulted in a higher conversion of volatiles.

To integrate the results and select the most promising autochthonous LAB, PCA was used to compare the performance of LAB inoculated in cheese models and Serpa PDO cheeses. Successive statistical analyses were performed to reduce the number of variables. A total of 21 attributes were selected as being discriminative, namely the quotient between WSN and TN and TCA and TN; the amino acids Glu, Ser, Ala, Val, Met, Tyr, Leu, Phe and total FAA; the VFA iC_5_, C_7_, C_9_, C_10_, and total VFA; the esters ethyl isovalerate, valerate, hexanoate and octanoate, and total esters. However, in the PCA projections, cheeses and cheese models were on the opposite side, since they are very different microenvironments, with distinct molecule concentration. However, conditions B (PL1), D (PL2) and C (PC and PL1) are aggregated or closer to Serpa PDO cheeses.

The same statistical analysis was also carried out including only cheese models. The quotient between WSN and TN, PTA and TN, and TCA and TN; the amino acids Glu, Ser, Ala, Val, Met, Tyr, Leu, and total FAA; the VFAs iC_5_, C_7_, C_12_, and total VFAs; and esters ethyl iso-valerate, valerate and octanoate, and total esters were selected as discriminative. Generally, conditions B, C, D and F are aggregated in all sample projections. Thus, both analyses suggested that the strains *L. plantarum* PL1, *L. plantarum* PL2 and the mix of *L. plantarum* PL1 and *L. paracasei* PC may be the most promising and result in a closer lipolytic and proteolytic profile of Serpa PDO cheese. This result aligns with some scientific studies that showed the importance of *L. plantarum* in the definition of final product characteristics.

In future work, these inocula will be produced at a pilot scale and tested at the cheese manufacturing level to validate their industrial application. A tailor-made starter culture contributes to higher quality and safety standards for raw milk cheeses, since it is never possible to have an exceptional microbiological quality for the PDO label. The raw milk with an exceptional biochemical profile can be directed for PDO certification, while the others can be directed for the application of autochthonous starter with or without pasteurization. Although this new product cannot be marketed with the PDO seal, it may have highly appreciated organoleptic characteristics and maintain some of the Serpa original product authenticity. The application of this starter culture will allow for the maximization of the small ruminant milk sector resources and reduce economic losses. At the same time, the pasteurization coupled with the starter culture addition will allow for the reaching of more restricted markets, where the use of raw milk is restricted or prohibited. On the other hand, in some European countries, the legislation has been updated, and the use of starter cultures for PDO cheeses is now accepted. This study may contribute to proposed changes in the applicable legislation to promote uniform quality, reduce losses and facilitate commercialization.

## Figures and Tables

**Table 1:** Summary of most relevant probiotic, safety, technological and protective properties of lactic acid bacteria (LAB) strains studied by Ruiz-Moyano et al. [14] and Araújo-Rodrigues et al. [22].

Species	Probiotic Potential [14]	Biogenic Amine Production and Antibiotic Resistance [14]	Extracellular Proteolytic Activity [22]	Lipolytic Activity [22]	Salt and Temperature Resistance [22]	D(-)-lactic acid Production [22] ^1^	Acidification Potential [22] ^2^	Antimicrobial Activity [22] ^3^
*Lacticaseibacillus paracasei* PC	-	-	+	+	+	<1	1.8–2.0	*Listeria monocytogenes* 934 *Salmonella choleraesuis* CECT 4395
*Lactiplantibacillus plantarum* PL1	+	-	+	+	+	6–8	2.6–2.8	3 *Listeria* spp. strains *Bacillus cereus* CECT 131 *S. choleraesuis* CECT 4395
*Lactiplantibacillus plantarum* PL2	+	-	-	-	+	8–10	2.6–2.8	3 *Listeria* spp. strains *B. cereus* CECT 131 *S. choleraesuis* CECT 4395
*Lactobacillus crustorum* CR	+	-	+	-	+	1–4	2.2–2.4	*S. choleraesuis* CECT 4395
*Lactobacillus pentosus* PE	+	-	+	-	+	6–8	2.6–2.8	*S. choleraesuis* CECT 4395
*Levilactobacillus brevis* BR1	+	-	+	-	+	1–4	2.0–2.2	*S. choleraesuis* CECT 4395

+ Activity detected; - Activity not detected; ^1^ g L^−1^; ^2^ Interval of pH variation after 24 h of inoculation. ^3^Antimicrobial inhibition higher than 20%. The codes in the Ruiz-Moyano et al. study [14] - A2LB1, G1Lb5, G2Lb9, A3Lb18, G4Lb18 and C1Lb21 correspond to PC, PL1, PL2, CR, PE and BR1 in the present study, respectively.

**Table 2:** pH evolution of cheese models during a ripening time of 45 days.

			Ripening Time		
Condition	0 Days	7 Days	15 Days	30 Days	45 Days
A: *L. paracasei PC*	5.81 ± 0.14 ^a^	5.19 ± 0.05 ^a^	4.96 ± 0.09 ^a^	4.70 ± 0.05 ^a^	4.62 ± 0.07 ^a^
B: *L. plantarum* PL1	5.88 ± 0.05 ^a^	4.80 ± 0.04 ^b^	4.65 ± 0.05 ^b^	4.38 ± 0.02 ^b,c^	4.31 ± 0.03 ^b,c^
C: *L. paracasei* PC and *L. plantarum* PL1	6.12 ± 0.00 ^b^	5.29 ± 0.03 ^a^	4.92 ± 0.14 ^a^	4.63 ± 0.10 ^a,c^	4.39 ± 0.07 ^c,d^
D: *L. plantarum* PL2	5.94 ± 0.13 ^a^	4.63 ± 0.03 ^c^	4.52 ± 0.02 ^b^	4.33 ± 0.02 ^b^	4.49 ± 0.08 ^a,d^
E: *L. crustorum* CR	5.73 ± 0.01 ^a^	4.64 ± 0.01 ^c^	4.52 ± 0.14 ^b^	4.42 ± 0.03 ^b,c^	4.23 ± 0.07 ^b^
F: *L. pentosus* PE	5.28 ± 0.04 ^c^	4.18 ± 0.04 ^d^	4.00 ± 0.03 ^c^	3.86 ± 0.02 ^d^	3.84 ± 0.01 ^e^
G: *L. brevis* BR1	6.22 ± 0.03 ^b^	5.77 ± 0.05 ^g^	5.33 ± 0.06 ^d^	5.22 ± 0.06 ^e^	4.98 ± 0.05 ^f^
H: Control	6.55 ± 0.01 ^d^	6.29 ± 0.01 ^f^	6.00 ± 0.01 ^e^	5.60 ± 0.02 ^f^	5.36 ± 0.03 ^g^

Different superscript letters in the same column (corresponding to the same ripening time) indicate significant differences between conditions (*p* < 0.05).

**Table 3:** The content (mean ± standard deviation) of free amino acids in cheese models (mg 100 g^−1^) incubated for 45 d.

				Condition				
FAA	A	B	C	D	E	F	G	H
Asp	68.10 ± 5.69 ^a,b^	58.69 ± 5.77 ^a,d^	73.14 ± 4.57 ^b^	159.67 ± 7.21 ^c^	39.10 ± 1.15 ^e^	49.10 ± 3.01 ^d,e^	26.08 ± 1.86 ^g^	13.49 ± 0.21 ^f^
Glu	126.88 ± 1.20 ^a^	105.57 ± 4.45 ^b^	150.89 ± 3.82 ^c^	164.47 ± 4.75 ^c^	127.52 ± 4.94 ^a^	80.52 ± 8.95 ^d^	46.70 ± 4.81 ^e^	78.55 ± 0.20 ^d^
Cys	N.D. ^a^	N.D. ^a^	1.30 ± 0.35 ^b^	5.03 ± 0.63 ^c^	6.25 ± 0.07 ^d^	N.D. ^a^	0.47 ± 0.02 ^a^	N.D. ^a^
Asn	31.44 ± 6.40 ^a^	28.39 ± 4.91 ^a^	31.56 ± 1.56 ^a^	42.88 ± 2.18 ^b^	18.12 ± 0.39 ^c,e^	28.09 ± 5.40 ^a,c^	13.93 ± 1.91 ^d^	5.18 ± 0.01 ^d^
Ser	52.22 ± 0.00 ^a^	7.04 ± 1.31 ^b^	22.53 ± 3.10 ^c^	17.72 ± 0.24 ^d^	8.34 ± 0.42 ^b,e^	11.51 ± 1.04 ^e^	15.60 ± 0.23 ^d^	5.79 ± 0.70 ^b^
Gln	117.14 ± 13.78 ^a^	30.55 ± 0.00 ^b^	103.22 ± 6.51 ^a,e^	11.78 ± 0.94 ^b,c^	N.D. ^c^	78.78 ± 22.41 ^e^	38.11 ± 6.55 ^d^	N.D. ^c^
Thr	37.61 ± 1.75 ^a^	12.97 ± 2.05 ^b^	27.53 ± 1.85 ^c^	25.99 ± 0.00 ^c^	14.83 ± 0.53 ^b^	18.69 ± 0.50 ^d^	5.96 ± 0.30 ^e^	N.D. ^f^
Arg	52.92 ± 6.86 ^a^	76.04 ± 3.33 ^b^	106.18 ± 5.87 ^c^	55.84 ± 5.75 ^a^	39.67 ± 1.37 ^d^	50.77 ± 5.68 ^a,d^	38.73 ± 1.12 ^d^	62.02 ± 0.50 ^a^
Ala	122.48 ± 1.44 ^a,b^	102.14 ± 0.44 ^c^	119.91 ± 5.49 ^a^	129.07 ± 1.35 ^b^	110.32 ± 0.18 ^c^	53.27 ± 5.05 ^d^	27.39 ± 1.93 ^e^	51.25 ± 2.57 ^d^
Tyr	109.32 ± 8.09 ^a^	65.03 ± 3.91 ^b^	105.37 ± 9.45 ^a^	60.84 ± 3.21 ^b^	1.50 ± 0.00 ^c^	N.D. ^c^	70.59 ± 5.97 ^b^	8.95 ± 0.59 ^c^
Val	80.58 ± 17.11 ^a^	30.55 ± 1.64 ^b^	57.01 ± 2.18 ^c^	108.11 ± 6.48 ^d^	68.17 ± 0.16 ^a,c^	65.87 ± 4.74 ^a,c^	53.48 ± 2.26 ^c^	9.46 ± 0.45 ^e^
Trp	58.49 ± 0.00 ^a^	N.D. ^b^	24.90 ± 4.95 ^c^	7.14 ± 1.11 ^d^	N.D. ^b^	16.02 ± 0.29 ^e^	22.28 ± 3.69 ^c,e^	N.D. ^b^
Phe	128.85 ± 11.45 ^a^	78.99 ± 4.99 ^d,e^	106.08 ± 6.86 ^b^	50.30 ± 3.28 ^f,g^	57.66 ± 0.19 ^e,f^	82.09 ± 8.89 ^c,d^	100.78 ± 9.26 ^b,c^	33.56 ± 3.73 ^g^
Ile	59.40 ± 0.90 ^a^	7.41 ± 0.94 ^b^	29.17 ± 2.52 ^c^	14.82 ± 1.34 ^d^	13.99 ± 0.38 ^d^	16.12 ± 3.09 ^d^	11.21 ± 2.23 ^b,d^	N.D. ^e^
Leu	362.85 ± 11.29 ^a^	240.14 ± 5.48 ^b^	259.76 ± 2.93 ^c^	257.44 ± 5.72 ^b,c^	121.31 ± 2.89 ^d^	167.21 ± 5.23 ^e^	194.98 ± 11.01 ^f^	49.74 ± 1.08 ^g^
T ^1^	1460.57 ± 43.53 ^a^	843.51 ± 12.84 ^b^	1218.54 ± 41.87 ^c^	1111.11 ± 23.76 ^d^	626.78 ± 4.69 ^e^	718.02 ± 22.88 ^f^	666.30 ± 24.32 ^e,f^	317.98 ± 7.46 ^g^

^1^ Total of detected and quantified FAA; N.D. Not detected. Different superscript letters in the same row indicate significant differences between the specific FAA in the conditions studied (*p* < 0.05). Conditions A—*L. paracasei* PC; B—*L. plantarum* PL1; C—*L. paracasei* PC and *L. plantarum* PL1; D—*L. plantarum* PL2; E—*L. crustorum* CR; F—*L. pentosus* PE; G—*L. brevis* BR1; and H—without inoculation.

**Table 4:** Content (mean ± standard deviation) of volatile fatty acids (VFA) in cheese models (mg 100 g^−1^), incubated for 45 d.

				Condition				
VFA	A	B	C	D	E	F	G	H
iC_5_	5.07 ± 0.45 ^a,b^	12.40 ± 2.47 ^c^	5.52 ± 1.29 ^a^	4.14 ± 0.08 ^a,b^	11.77 ± 1.26 ^c^	2.12 ± 0.53 ^b,d^	0.28 ± 0.02 ^d^	0.36 ± 0.01 ^d^
C_5_	4.74 ± 0.41 ^a,b^	4.28 ± 0.90 ^a^	43.94 ± 0.62 ^c^	7.73 ± 0.40 ^b,d^	10.58 ± 2.99 ^d^	0.27 ± 0.02 ^e^	0.71 ± 0.15 ^e^	0.35 ± 0.09 ^e^
C_6_	679.33 ± 5.85 ^a,b^	230.47 ± 52.62 ^c,d^	712.74 ± 14.44 ^a,b^	826.58 ± 144.78 ^b^	1689.07 ± 221.27 ^e^	515.68 ± 126.03 ^a,d^	8.78 ± 0.23 ^c^	26.09 ± 2.94 ^c^
C_7_	11.94 ± 1.47 ^a^	10.95 ± 1.69 ^a^	11.85 ± 0.42 ^a^	16.53 ± 1.28 ^a^	28.63 ± 5.52 ^b^	15.60 ± 0.26 ^a^	1.15 ± 0.27 ^c^	0.31 ± 0.05 ^c^
C_8_	736.15 ± 78.56 ^a.b^	555.73 ± 80.34 ^a^	697.15 ± 6.72 ^a,b^	666.96 ± 113.86 ^a,b^	1403.49 ± 185.82 ^c^	910.45 ± 19.95 ^b^	90.76 ± 9.08 ^d^	21.03 ± 0.43 ^d^
C_9_	9.41 ± 2.51 ^a,b,c^	6.78 ± 1.54 ^c,d^	8.84 ± 0.69 ^b,c,d^	7.12 ± 2.09 ^c,d^	15.53 ± 4.31 ^a^	14. 51 ± 2.42 ^a,b^	3.12 ± 0.83 ^d^	2.72 ± 0.16 ^d^
C_10_	742.24 ± 8.83 ^a,b,c^	601.01 ± 153.83 ^a^	609.91 ± 22.82 ^a,b^	882.07 ± 133.13 ^c^	716.96 ± 139.11 ^a,b,c^	861.11 ± 43.09 ^b,c^	203.22 ± 43.59 ^d^	16.57 ± 2.04 ^d^
C_12_	77.27 ± 12.43 ^a,b^	47.24 ± 4.04 ^a^	61.34 ± 1.79 ^a,b^	70.40 ± 11.21 ^a,b^	73.72 ± 18.16 ^a,b^	89.51 ± 0.58 ^b^	72.63 ± 17.83 ^a^	2.43 ± 0.37 ^c^
Total ^1^	2266.15 ± 92.85 ^a^	1468.87 ± 18.48 ^b^	2151.28 ± 44.13 ^a,b^	2481.52 ± 402.64 ^a^	3949.74 ± 578.44 ^c^	2409.24 ± 145.39 ^a^	380.65 ± 69.29 ^d^	69.86 ± 5.60 ^d^

^1^ Total of detected and quantified VFA; N.D. Not detected. Different superscript letters in the same row indicate significant differences between conditions (*p* < 0.05). Conditions A—*L. paracasei* PC; B—*L. plantarum* PL1; C—*L. paracasei* PC and *L. plantarum* PL1; D—*L. plantarum* PL2; E—*L. crustorum* CR; F—*L. pentosus* PE; G—*L. brevis* BR1; and H—without inoculation.

**Table 5:** Content (mean ± standard deviation) of esters in cheese models (mg 100 g^−1^), incubated for 45 d.

				Condition				
Ester	A	B	C	D	E	F	G	H
Ethyl butanoate	28.86 ± 4.68 ^a^	7.36 ± 2.05 ^b^	6.06 ± 0.72 ^b^	23.27 ± 3.54 ^a^	29.40 ± 3.05 ^a^	11.08 ± 1.19 ^b^	67.31 ± 7.57 ^c^	6.97 ± 0.86 ^b^
Ethyl isovalerate	N.D. ^a^	N.D. ^a^	N.D. ^a^	N.D. ^a^	N.D. ^a^	N.D. ^a^	0.50 ± 0.07 ^b^	N.D. ^a^
Ethyl valerate	0.36 ± 0.10 ^a^	0.59 ± 0.02 ^a^	0.15 ± 0.02 ^a^	0.26 ± 0.06 ^a^	N.D. ^a^	0.15 ± 0.01 ^a^	4.85 ± 0.78 ^b^	N.D. ^a^
Ethyl hexanoate	34.89 ± 9.08 ^a,b^	10.18 ± 0.46 ^c,d^	13.65 ± 2.20 ^c,d^	23.13 ± 2.37 ^a,c^	35.80 ± 5.42 ^a,b^	16.24 ± 3.38 ^c,d^	49.15 ± 8.16 ^b^	7.70 ± 2.27 ^d^
Ethyl Heptanoate	1.48 ± 0.18 ^a,b^	0.85 ± 0.13 ^a,c^	0.98 ± 0.18 ^a^	0.62 ± 0.04 ^a,c^	2.15 ± 0.36 ^b^	1.06 ± 0.27 ^a^	9.27 ± 0.72 ^d^	N.D. ^c^
Ethyl Octanoate	82.75 ± 24.68 ^a,b^	28.25 ± 6.44 ^b,c^	15.16 ± 4.61 ^c^	18.83 ± 5.19 ^c^	116.67 ± 30.03 ^a^	66.90 ± 22.04 ^a,b,c^	445.79 ± 32.71 ^d^	18.28 ± 4.51 ^c^
Ethyl Nonanoate	1.17 ± 0.21 ^a^	0.30 ± 0.06 ^a^	0.16 ± 0.00 ^a^	0.73 ± 01 ^a^	1.28 ± 0.13 ^a^	0.82 ± 0.21 ^a^	16.57 ± 2.69 ^b^	0.24 ± 0.05 ^a^
Ethyl decanoate	166.50 ± 13.11 ^a^	24.61 ± 5.77 ^a^	26.34 ± 6.42 ^a^	27.97 ± 4.01 ^a^	128.24 ± 35.00 ^a^	77.55 ± 21.91 ^a^	1954.83 ± 135.72 ^b^	27.64 ± 4.06 ^a^
Ethly dodecanoate	68.30 ± 3.56 ^a^	26.34 ± 7.59 ^a^	35.93 ± 8.36 ^a^	22.33 ± 4.00 ^a^	107.83 ± 10.97 ^a^	49.04 ± 13.40 ^a^	878.09 ± 145.55 ^b^	6.05 ± 0.23 ^a^
Total ^1^	384.32 ± 48.46 ^a^	98.46 ± 22.52 ^b,c^	98.43 ± 20.70 ^b,c^	117.15 ± 19.22 ^b,c^	421.37 ± 84.96 ^a^	222.83 ± 62.41 ^b^	3426.36 ± 43.08 ^d^	66.88 ± 10.15 ^c^

^1^ Total of detected and quantified ester; N.D. Not detected. Different superscript letters in the same line indicate significant differences between conditions (*p* < 0.05). Conditions A—*L. paracasei* PC; B—*L. plantarum* PL1; C—*L. paracasei* PC and *L. plantarum* PL1; D—*L. plantarum* PL2; E—*L. crustorum* CR; F—*L. pentosus* PE; G—*L. brevis* BR1; and H—without inoculation.

## Data Availability

The data is included in the article.
